# The *Atoh1-Cre* Knock-In Allele Ectopically Labels a Subpopulation of Amacrine Cells and Bipolar Cells in Mouse Retina

**DOI:** 10.1523/ENEURO.0307-23.2023

**Published:** 2023-11-02

**Authors:** Sih-Rong Wu, Huda Y. Zoghbi

**Affiliations:** 1 Department of Neuroscience, Baylor College of Medicine, Houston, Texas 77030; 2 Jan and Dan Duncan Neurological Research Institute, Texas Children’s Hospital, Houston, Texas 77030; 3 Department of Molecular and Human Genetics, Baylor College of Medicine, Houston, Texas 77030; 4 Department of Pediatrics, Baylor College of Medicine, Houston, Texas 77030; 5 Howard Hughes Medical Institute, Baylor College of Medicine, Houston, Texas 77030

**Keywords:** amacrine cells, *Atoh1*, bipolar cells, ectopic Cre expression, knock-in mouse model, retina

## Abstract

The retina has diverse neuronal cell types derived from a common pool of retinal progenitors. Many molecular drivers, mostly transcription factors, have been identified to promote different cell fates. In *Drosophila*, *atonal* is required for specifying photoreceptors. In mice, there are two closely related *atonal* homologs, *Atoh1* and *Atoh7*. While *Atoh7* is known to promote the genesis of retinal ganglion cells, there is no study on the function of *Atoh1* in retinal development. Here, we crossed *Atoh1^Cre/+^* mice to mice carrying a Cre-dependent TdTomato reporter to track potential *Atoh1*-lineage neurons in retinas. We characterized a heterogeneous group of TdTomato^+^ retinal neurons that were detected at the postnatal stage, including glutamatergic amacrine cells, AII amacrine cells, and BC3b bipolar cells. Unexpectedly, we did not observe TdTomato^+^ retinal neurons in the mice with an *Atoh1-FlpO* knock-in allele and a Flp-dependent TdTomato reporter, suggesting *Atoh1* is not expressed in the mouse retina. Consistent with these data, conditional removal of *Atoh1* in the retina did not cause any observable phenotypes. Importantly, we did not detect *Atoh1* expression in the retina at multiple ages using mice with *Atoh1-GFP* knock-in allele. Therefore, we conclude that *Atoh1^Cre/+^* mice have ectopic Cre expression in the retina and that *Atoh1* is not required for retinal development.

## Significance Statement

The *Atoh1^Cre/+^* mice have been broadly used to study the *Atoh1*-lineage cells in many different contexts including neurons in the hindbrain and the spinal cord, hair cells in the inner ear, secretory cells in the intestine, Merkel cells in the epidermis, and tumor cells in the medulloblastoma. While the *Atoh1-Cre* allele matches endogenous *Atoh1* expression in these tissues, in this study we describe ectopic expression of *Atoh1-Cre* allele in the retina and demonstrate that *Atoh1* is not required for retinal development. Importantly, we further characterized the cell types that were reliably labeled by the ectopic Cre, providing a potential tool to target retinal cells with *Atoh1^Cre/+^* mouse model.

## Introduction

The retina is a thin layer of tissue that converts the light to the electrical signal and transmits the information to the visual system in the brain. There are six major neuronal cell types, as follows: rod and cone photoreceptors; bipolar, horizontal, and amacrine interneurons; retinal ganglion cells; and a glial cell type, Müller glia, in the mammalian retina. These cells are derived from a pool of retinal progenitor cells (Turner and Cepko, 1987). Recently, single-cell RNA sequencing (RNA-seq) uncovered >60 neuronal subtypes in the retina (Peng et al., 2019; Yan et al., 2020), raising an important question about how retinal cell diversity arises during development. The basic helix-loop-helix (bHLH) transcription factor *atonal* (*ato*) plays a key role in the developing retina in *Drosophila* by promoting the transition from retinal progenitor cells to photoreceptors (Jarman et al., 1994). Among the seven mammalian homologs of *ato*, only ATOH1 and ATOH7 share the identical basic domain with ATO, which is important for DNA binding (Hassan and Bellen, 2000). In contrast to *ato* in the fruit fly, *Atoh7* is required for genesis of retinal ganglion cells rather than photoreceptors in mice (Brown et al., 2001). *Atoh1*, on the other hand, has not been studied in mammalian retinas yet.

Mouse genetic tools have been used to interrogate the function of *Atoh1* by replacing the coding region of *Atoh1* with either a lacZ reporter or a Cre recombinase, which allows us to remove *Atoh1* and track the *Atoh1*-lineage neurons in combination with a Cre-dependent reporter (Ben-Arie et al., 2000; Yang et al., 2010). The specificity of labeling *Atoh1* lineage using lacZ reporter or Cre recombinase has been validated by loss of the labeled cells upon *Atoh1* deletion. Specifically, *Atoh1* knockout leads to loss of several neuronal cell types in the brainstem, granule cells in the cerebellum, interneurons in the spinal cord (Hassan and Bellen, 2000; Bermingham et al., 2001; Wang et al., 2005; Rose et al., 2009), as well as non-neuronal cells such as Merkel cells in the skin (Morrison et al., 2009), secretory cells in the intestine (Yang et al., 2001), and hair cells in the inner ear (Yang et al., 2010). Although *Atoh1*-lineage neurons have been systematically identified in the mouse hindbrain and shown to be critical components of the auditory, vestibular, proprioceptive, and interoceptive pathways (Rose et al., 2009), it remains unclear whether *Atoh1* is also involved in retinal development.

In this study, we set out to characterize the potential *Atoh1*-lineage neurons in the mouse retina by crossing *Atoh1^Cre/+^* mice to the mice carrying a Cre-dependent TdTomato reporter (Ai14). While we detected TdTomato labeling in the retina by *Atoh1-Cre* at the postnatal stage, further studies using *Atoh1^FlpO^*^/+^ mice with a Flp-dependent reporter (Ai65F) revealed that the *Atoh1-Cre* allele leads to ectopic expression in the retina. Interestingly, the ectopic labeling is consistent across animals and enriched for vesicular glutamate transporter 3^+^ (VGluT3^+^) amacrine cells, AII amacrine cells, and BC3b bipolar cells. Last, we demonstrated that conditional knockout (cKO) of *Atoh1* in mouse retina does not cause histologic phenotype, suggesting that *Atoh1* does not play a role in retinal development.

## Materials and Methods

### Animals

The following mouse lines were used in this study: *Atoh1^lacZ/+^* (catalog #005970, The Jackson Laboratory); *Atoh1^Cre/+^* (Yang et al., 2010); *Atoh1^FlpO^*^/+^ (catalog #036541, The Jackson Laboratory); *Atoh1^flox/+^* (catalog #008681, The Jackson Laboratory); *mRx-Cre* (Klimova et al., 2013); Ai65 (catalog #021875, The Jackson Laboratory); and Ai14 (catalog #007914, The Jackson Laboratory). Ai65F mice were generated by crossing the Ai65 mice to *Sox2-Cre* mice (catalog #008454, The Jackson Laboratory). All mice were housed in a level 3, American Association for Laboratory Animal Science-certified facility on a 14 h light/dark cycle. Husbandry, housing, killing, and experimental guidelines were approved by the institutional animal care and use committee at Baylor College of Medicine.

### Immunofluorescence staining

After the euthanasia and enucleation of the animals, the corneas and the lenses were removed from the globes. Both males and females were collected. The globes were fixed with 4% paraformaldehyde in PBS for 30 min on ice, followed by PBS wash and incubation in 30% sucrose in PBS at 4°C for 14–16 h. The samples were cryopreserved in optimal cutting temperature (OCT) compound and stored at −80°C until use. The retinal sections were collected on slides by cryostat (Leica) with 10–20 μm thickness and stored at −20°C until use. For immunofluorescence staining, the slides were rinsed with PBS to remove OCT, followed by blocking with the blocking buffer (5% normal goat serum with 0.2% Triton X-100 in PBS) for 1 h at room temperature (RT). The sections were incubated with primary antibodies in blocking buffer at 4°C for 24 h, followed by PBS wash for three times. The sections were incubated with secondary antibodies in blocking buffer at RT for 2 h. The counterstain was performed by DAPI staining at RT for 5 min. The slides were mounted with Vectashield Antifade Mounting Media (catalog #H-1000–10, Vector Laboratories). The following primary antibodies were used in this study with the following indicated dilutions: anti-SLC6A9 (GlyT1; 1:1000; catalog #BMP091, MBL); anti-GAD65/67 (1:500; catalog #AB1511, Millipore); anti-Pax6 (1:500; catalog #901301, BioLegend); anti-TH (1:500; catalog #AB152, Millipore); anti-ChAT (1:100; catalog #AB144P, Millipore); anti-VGluT3 (1:2000; catalog #AB5421-1, Millipore); anti-Dab1 (1:200; catalog #AB5840-1, Millipore); anti-Vsx2 (1:200; catalog #X1179P, Exalpha); anti-Islet1/2 (1:100; catalog #40.2D6, Developmental Studies Hybrbridoma Bank); anti-Prkar2b (1:500; catalog #610625, BD); anti-PKCα (1:100; catalog #610107, BD); anti-Hcn4 (1:500; catalog #APC-052, Alomone Labs); anti-Syt2b (1:200; catalog #ZDB-ATB-081002–25, ZFIN); and anti-GFP (1:1000; catalog #ab13970, Abcam).

### Quantification of the immunostaining and statistics

For colocalization studies, three animals were used. Six regions of interest were quantified for each animal. The total number of marker^+^ cells and the number of marker^+^ and TdTomato^+^ cells were determined by manually counting with ImageJ. The percentage of marker^+^ cells overlapped with TdTomato was presented as the mean ± SD for each retinal subtype. For *Atoh1* cKO studies, three animals were used. Six regions of interest were quantified for each animal. The total number of marker^+^ cells were determined by manually counting with ImageJ. Data were presented as the mean ± SD for each retinal subtype. Statistics were performed between the control and *Atoh1* cKO using the *t* test.

## Results

### Genetic lineage tracing indicates *Atoh1-Cre* labels several neurons postnatally in the mouse retina

**Figure 1.**
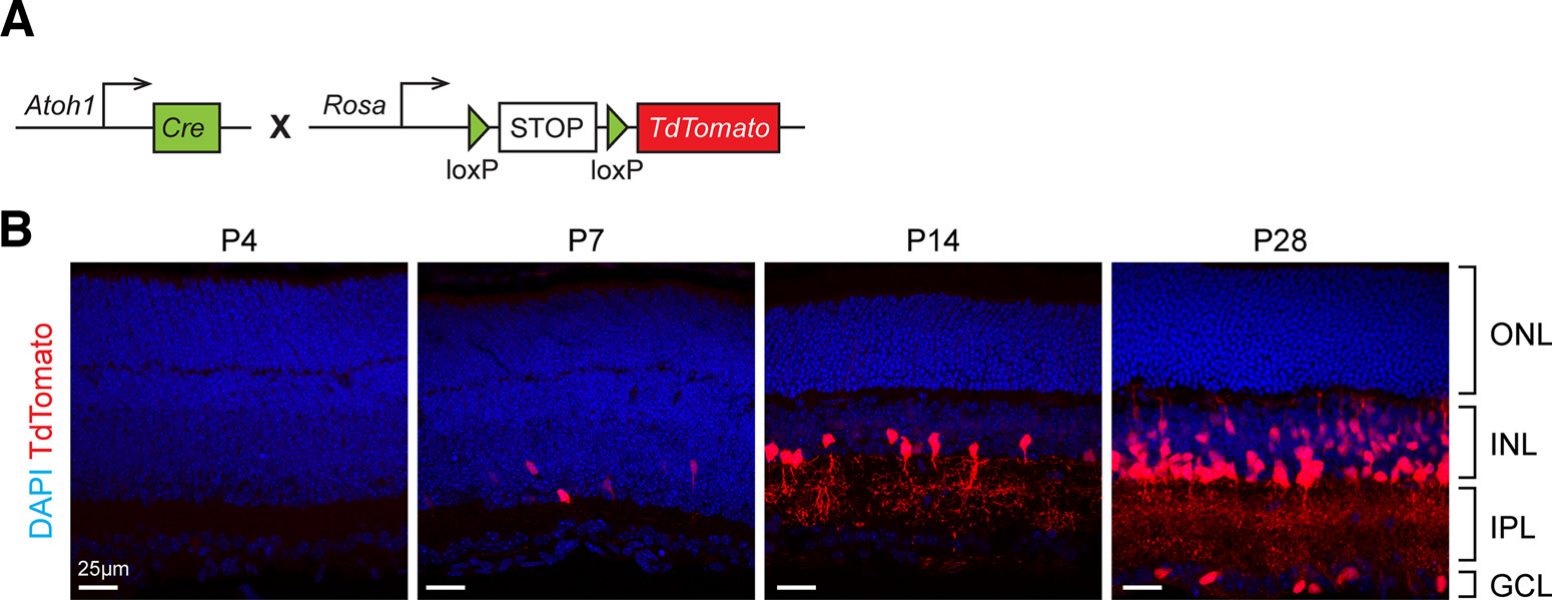
Genetic lineage tracing reveals *Atoh1-Cre*-labeled neurons in the mouse retina. ***A***, A schematic illustration of the lineage-tracing method. *Atoh1-Cre* knock-in mice (*Atoh1^Cre/+^*) were crossed with mice carrying a Cre-dependent TdTomato reporter (Ai14). ***B***, TdTomato expression in the mouse retina at different postnatal stages. The retinas were collected from *Atoh1^Cre/+^; Ai14/+* mice at P4, P7, P14, and P28 (*n* = 3 per timepoint). The nuclei were stained with DAPI.

To test whether *Atoh1* contributes to retinal cells, we crossed *Atoh1^Cre/+^* mice (Yang et al., 2010) to Ai14 reporter mice (*Rosa^lsl-TdTomato^*; Madisen et al., 2010; Fig. 1*A*). We examined the mouse retinas at different timepoints to determine whether there are TdTomato^+^ cells (presumably *Atoh1*-lineage cells) and, if so, where and when those cells emerge. We did not find TdTomato^+^ cells until postnatal day 7 (P7). We found a few TdTomato^+^ cells in the inner nuclear layer (INL) at P7 (Fig. 1*B*). The number of TdTomato^+^ cells increased as the animals aged. At P14, most of the TdTomato^+^ cells were located at the inner part of INL, where most amacrine cells reside (Fig. 1*B*). In addition, TdTomato labeling extended to the inner plexiform layer (IPL), where amacrine cells project their dendritic arborization. At P28, TdTomato^+^ cells were observed in the outer layer of INL, the area occupied by bipolar cells and horizontal cells, and in the ganglion cell layer (GCL), the region where ganglion cells and amacrine cells reside (Fig. 1*B*). We did not find TdTomato^+^ cells in the outer nuclear layer (ONL), suggesting that *Atoh1* did not contribute to rod and cone photoreceptors (Fig. 1*B*). Together, these data suggest that *Atoh1* may be expressed at the postnatal stages and give rise to a subset of retinal neurons, including amacrine cells, bipolar cells, horizontal cells, and ganglion cells.

### *Atoh1-Cre*-labeled neurons include glutamatergic amacrine cells, AII amacrine cells, and BC3b OFF bipolar cells

**Figure 2.**
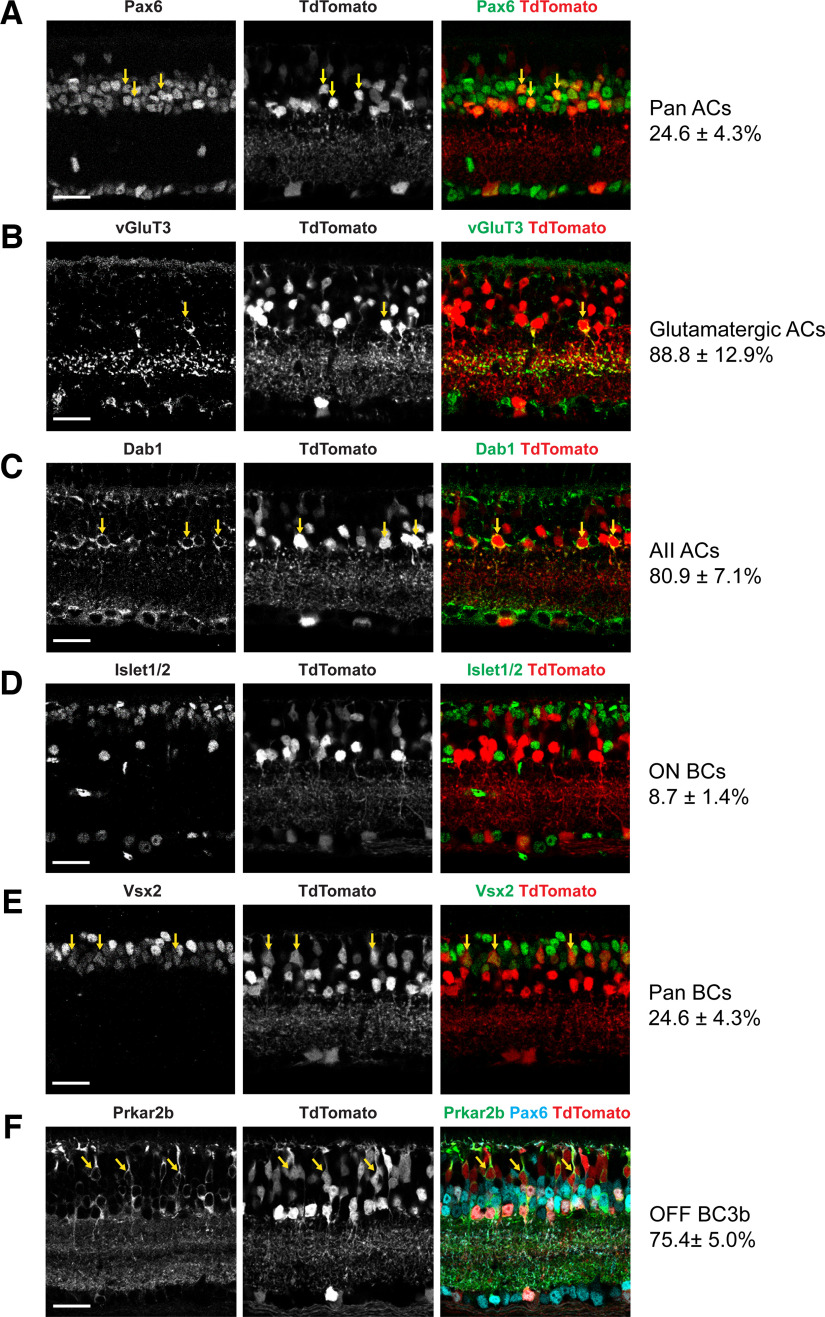
*Atoh1^Cre/+^* labels a heterogeneous group of neurons in the retina. ***A***–***F***, Immunofluorescence staining of different retinal cell markers on adult mouse retina. The retinas were collected from *Atoh1^Cre^*^/+^*; Ai14*/*+* mice. The arrows show examples of the colocalization of the marker and TdTomato. For BC3b subtype (***F***), Pax6 was used as an additional marker to exclude Prkar2b^+^ amacrine cells. The percentage of the marker^+^ cells overlapping with TdTomato is calculated and reported as the mean ± SD (*n* = 3 for each marker). Scale bar, 25 μm. AC, Amacrine cell; BC, bipolar cell. Also see Extended Data Figures 2-1 and 2-2 for additional immunofluorescence staining for ACs and BCs, respectively. The quantification of AC and BC subtypes was summarized in Extended Data Tables 2-1 and 2-2, respectively.

Given the heterogeneity of the retinal neurons, we sought to characterize the TdTomato^+^ neuronal subtypes labeled by *Atoh1-Cre* using immunofluorescence staining in adult mice with subtype-specific markers. The percentage of the marker^+^ cells that coexpress TdTomato was calculated. We focused on amacrine cells and bipolar cells because these two cell types compose the majority of the TdTomato^+^ cells based on their anatomic location. First, we examined the amacrine cells, the interneurons in INL and GCL, which are categorized into the following two broad groups: glycinergic amacrine cells and GABAergic amacrine cells (Extended Data Table 2-1). Using a pan amacrine cell marker Pax6, we observed 29.0 ± 3.1% of amacrine cells are *Atoh1-Cre*-labeled neurons (Fig. 2*A*). In addition, we found 67.4 ± 6.7% of glycinergic amacrine cells labeled by glycine transporter 1^+^ (GlyT1^+^) are TdTomato^+^ (Extended Data Fig. 2-1*A*). On the other hand, ∼44.6 ± 2.9% of GABAergic amacrine cells demarcated by glutamate decarboxylase (GAD65/67^+^) are TdTomato^+^ (Extended Data Fig. 2-1*B*). Both glycinergic and GABAergic amacrine cells can be classified into subtypes depending on the expression of other neurotransmitters or neuropeptides (Extended Data Table 2-1). Interestingly, the glycinergic amacrine cells coexpressing glutamate are highly overlapped with TdTomato (88.8 ± 12.9%) shown by vesicular glutamate transporter 3 (VGluT3) staining (Fig. 2*B*). Moreover, the AII amacrine cell subtype defined by Dab1 expression also exhibits a high percentage of overlapping with TdTomato (80.9 ± 7.1%; Fig. 2*C*). In contrast, we detected no GABAergic amacrine cells coexpressing dopamine that are TdTomato^+^ and only a few GABAergic amacrine cells coexpressing acetylcholine that are TdTomato^+^ (1.7 ± 1.8%; Extended Data Fig. 2-1*C*,*D*). These data suggest that *Atoh1* may contribute to other GABAergic subtypes that express either neuropeptide Y or nitric oxide.

10.1523/ENEURO.0307-23.2023.f2-1 Figure 2-1 Characterization of TdTomato^+^ amacrine subtypes. ***A–D***, Immunofluorescence staining of different markers for amacrine subtypes on adult mouse retinas. The retinas were collected from *Atoh1^Cre^*^/+^*; Ai14*/*+* mice. The arrows show examples of the colocalization of the marker and TdTomato. The percentage of the marker^+^ cells overlapping with TdTomato is calculated and reported as the mean ± SD (*n* = 3 for each marker). Scale bar, 25 μm. ACs, Amacrine cells. Download Figure 2-1, TIF file.

10.1523/ENEURO.0307-23.2023.f2-2 Figure 2-2 Characterization of TdTomato^+^ bipolar subtypes. ***A–C***, Immunofluorescence staining of different markers for amacrine subtypes on adult mouse retina. The retinas were collected from *Atoh1^Cre/+^; Ai14/+* mice. The arrows show examples of the colocalization of the marker and TdTomato. For BC3a subtype (***B***), Pax6 was used as an additional marker to exclude Hcn4^+^ amacrine cells, shown by the yellow arrow. In contrast, the white arrowhead denotes the BC3a neuron that was not labeled by TdTomato. The percentage of the marker^+^ cells overlapping with TdTomato is calculated and reported as the mean ± SD (*n* = 3 for each marker). Scale bar, 25 μm. BC, Bipolar cell. Download Figure 2-2, TIF file.

10.1523/ENEURO.0307-23.2023.tab2-1 Extended Data Table 2-1 Characterization the TdTomato^+^ amacrine cells in *Atoh1^Cre/+^; Ai14/+* mice. Download Table 2-1, DOC file.

10.1523/ENEURO.0307-23.2023.tab2-2 Extended Data Table 2-2 Characterization the TdTomato^+^ bipolar cells in *Atoh1^Cre/+^; Ai14/+* mice. Download Table 2-2, DOC file.

Next, we examined the bipolar cells, the key neurons that are located in INL and directly receive inputs from the photoreceptors and transmit the signals to the ganglion cells. There are >10 subtypes of bipolar cells, which are divided into two broad categories, ON and OFF bipolar cells, depending on their chromatic preference. Several markers have been used to identify different subtypes (Cheng et al., 2005; Wässle et al., 2009). For example, Islet-1 is selectively expressed in ON, but not in OFF, bipolar cells. We found that <10% of ON bipolar cells were TdTomato^+^ (8.7 ± 1.4%; Fig. 2*D*). Using PKCα as a marker, we observed 4.3 ± 2.0% of rod bipolar cells are TdTomato^+^ (Extended Data Fig. 2-2*A*). However, when we stained for Vsx2, a pan marker for bipolar cells, we observed that about one-quarter of bipolar cells also express TdTomato (24.6 ± 4.3%; Fig. 2*E*), suggesting that *Atoh1-Cre* was expressed in OFF bipolar cells. Therefore, we used the following markers Syt2b, Hcn4, and Prkar2b (PKA type II β regulatory subunit) to identify BC2, BC3a, and BC3b OFF bipolar cells, respectively (Extended Data Table 2-2). While none of the BC3a neurons coexpressed TdTomato, 75.4 ± 5.0% of BC3b bipolar cells were labeled with TdTomato (Fig. 2*F*, Extended Data Fig. 2-2*B*). In addition, a small population of BC2 coexpressed TdTomato (3.7 ± 3.8%; Extended Data Fig. 2-2*C*).

Altogether, these data demonstrated that *Atoh1^Cre/+^* labels a heterogeneous group of neurons in the retina consistently across adult mice. In addition, we characterized several retinal subtypes that are highly overlapped with TdTomato, including glutamatergic amacrine cells, AII amacrine cells, and OFF bipolar cell subtype BC3b.

### *Atoh1*-deficient mice do not exhibit cellular phenotypes in the retina

**Figure 3.**
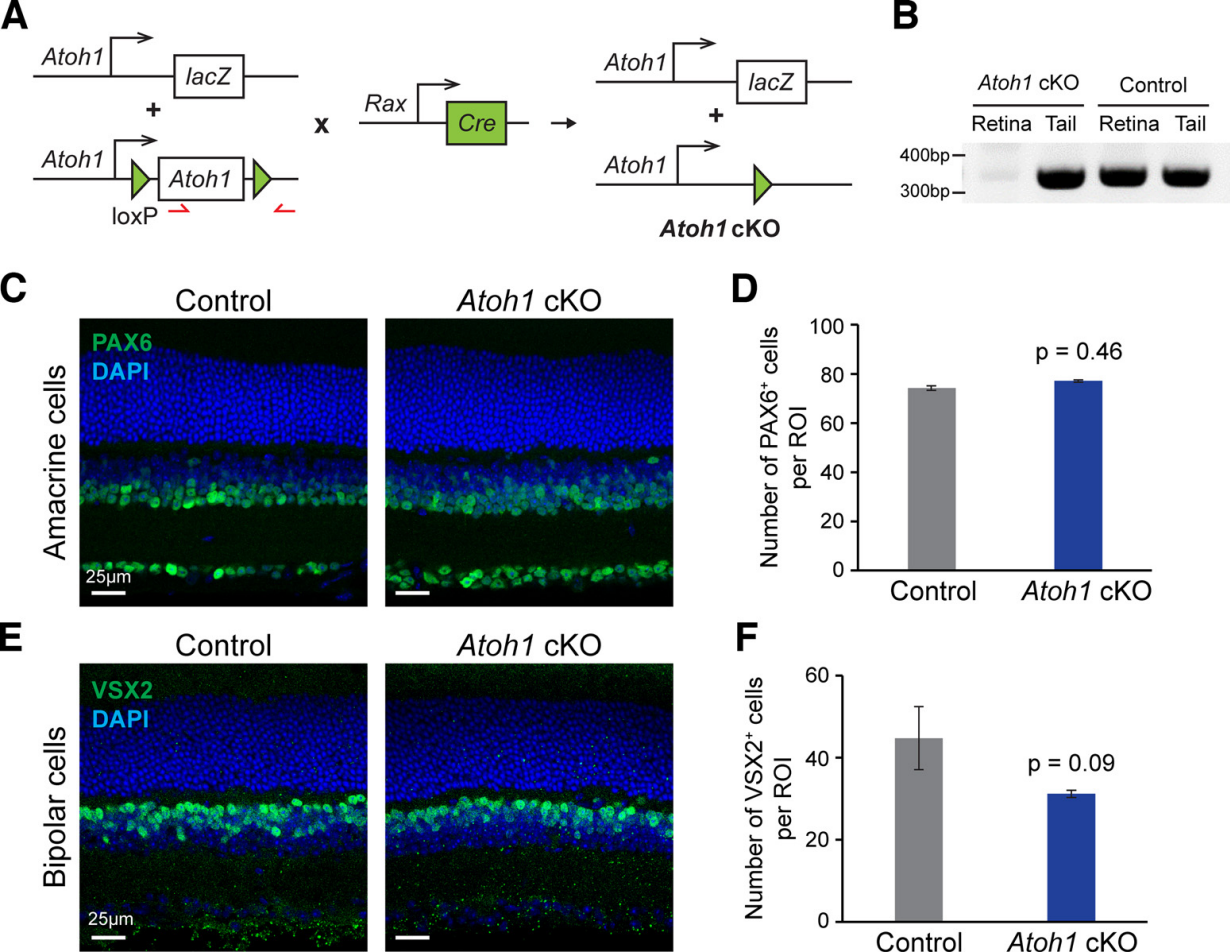
Conditional removal of *Atoh1* in the retina does not cause cellular phenotypes. ***A***, A schematic illustration of the strategy to knock out *Atoh1* specifically in mouse retina. ***B***, The red arrows denote the primers used to detect knockout efficiency. ***B***, PCR validation of *Atoh1* knockout in the retina. The genomic DNA was extracted from the retina and the tail of the control and *Atoh1* cKO mice, respectively. PCR was performed using the primers shown in ***A***. ***C***, ***E***, Immunofluorescence staining of PAX6 (***C***) and VSX2 (***E***) on the retinas from control and *Atoh1* cKO mice (*n* = 3 per genotype). The nuclei were stained with DAPI. ***D***, ***F***, Quantification of the PAX6^+^ (***D***) and VSX2^+^ (***F***) neurons in control and *Atoh1* cKO mice. Data are presented as the mean ± SD (*n* = 3 per genotype). The *p* values were determined by the *t* test (Extended Data Fig. 3-1, additional immunofluorescence staining and quantification).

To test whether *Atoh1* is required for development of the *Atoh1-Cre*-labeled neurons in the retina, we conditionally deleted *Atoh1* in the retina using *mRx-Cre* transgenic mice (Klimova et al., 2013). To ensure the *Atoh1* knockout efficiency, we used mice carrying an *Atoh1^flox^* allele (Shroyer et al., 2007) and an *Atoh1^lacZ^* allele (Ben-Arie et al., 2000), in which the coding region of *Atoh1* was replaced with *lacZ* allele, creating a null allele (Fig. 3*A*). We confirmed the conditional knockout in the retinal cell but not in other tissues using PCR (Fig. 3*B*) and quantified the numbers of amacrine cells and bipolar cells in control and *mRx-Cre; Atoh1^flox/lacZ^* mice (*Atoh1* cKO), respectively. There is no significant difference in the number of total amacrine cells (Pax6^+^) or bipolar cells (Vsx2^+^) between the control and *Atoh1* cKO mice (Fig. 3*C–F*). Moreover, we also found no change in the numbers of the subtypes including glycinergic amacrine cells (GlyT1^+^), glutamatergic amacrine cells (VGluT3^+^), BC3b OFF bipolar cells (Prkar2b^+^), and rod ON bipolar cells (PKCα^+^; Extended Data Fig. 3-1). Overall, we did not find a significant difference in the gross morphology of the retina and the number of major retinal cell types between control and *Atoh1* cKO mice. These data suggest that *Atoh1* is not required for the generation of the *Atoh1-Cre*-labeled cells in retina. However, we cannot exclude the possibility that the function of the potential *Atoh1*-lineage cells is impaired. Moreover, without labeling the *Atoh1*-lineage neurons with fluorescence reporter, the phenotype, if any, could be masked by the non *Atoh1*-lineage neurons.

10.1523/ENEURO.0307-23.2023.f3-1 Figure 3-1 *Atoh1* conditional knockout in the retina does not cause cellular phenotypes. ***A***, ***C***, ***E***, ***G***, Immunofluorescence staining of cell type-specific markers on the retinas from control and *Atoh1* cKO mice (*n* = 3 per genotype). The nuclei were stained with DAPI. For BC3b subtype (***E***), Pax6 was used as an additional marker to exclude Prkar2b^+^ amacrine cells. ***B***, ***D***, ***F***, ***H***, Quantification of the retinal subtypes in control and *Atoh1* cKO mice. Data are presented as the mean ± SD (*n* = 3 per genotype). The *p* values were determined by *t* test. Download Figure 3-1, TIF file.

### Ectopic expression of Cre recombinase under *Atoh1* promoter labels a subpopulation of retinal cells

**Figure 4.**
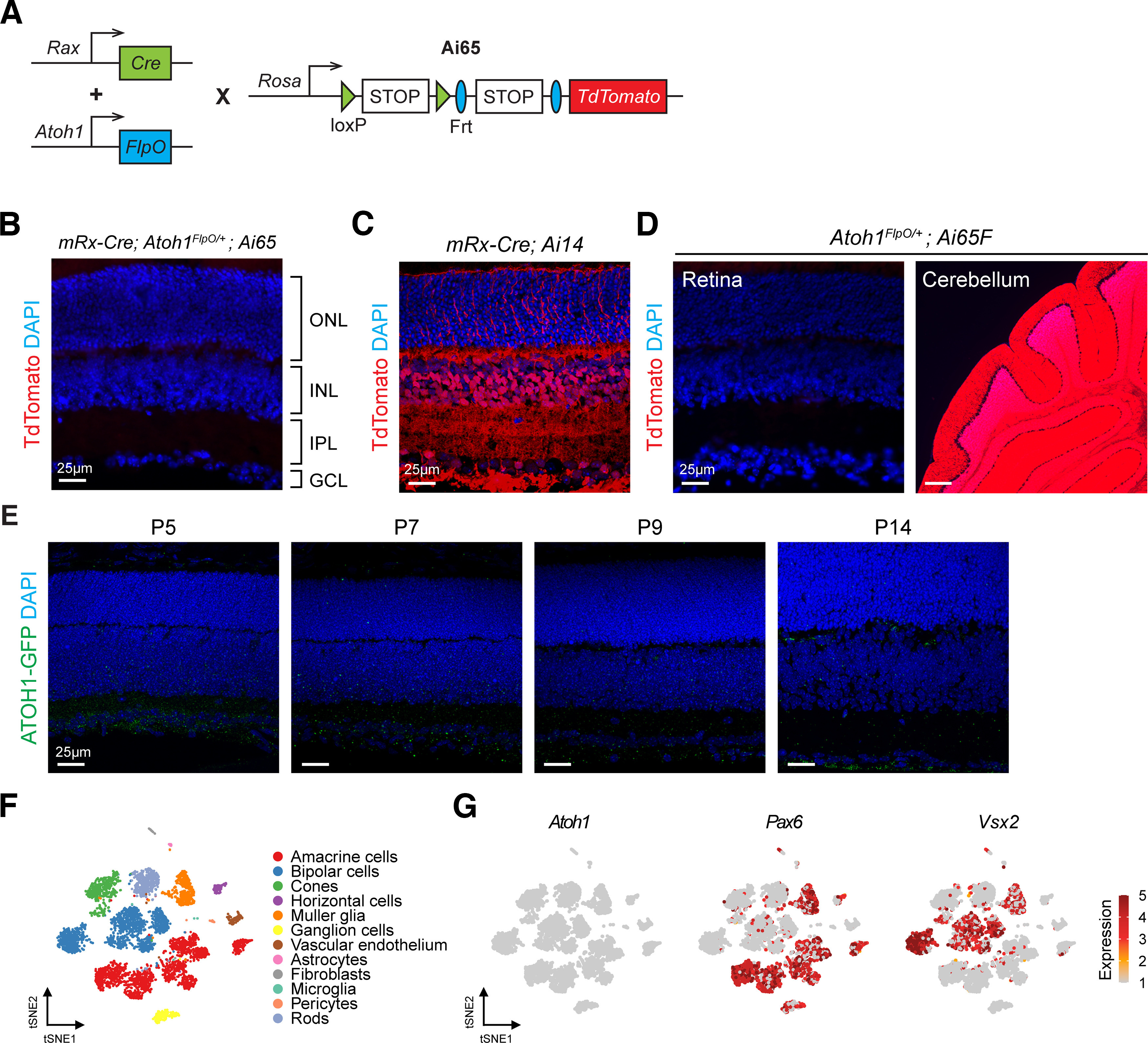
*Atoh1-Cre* but not *Atoh1-FlpO* lead to ectopic expression of TdTomato^+^ cells in the retina. ***A***, A schematic illustration of the intersectional labeling approach. *mRx-Cre*; *Atoh1^FlpO^*^/+^ mice were crossed to mice carrying a TdTomato reporter (Ai65) whose expression requires both Cre and Flp recombinases. ***B***, TdTomato expression in the retinas of *mRx-Cre*; *Atoh1^FlpO^*^/+^*; Ai65*/+ mice. The retinas were collected at P28 (*n* = 3). The nuclei were stained with DAPI. ***C***, Validation of TdTomato expression in the retinas of *mRx-Cre; Ai14/+* mice. The retinas were collected at P28 (*n* = 3). The nuclei were stained with DAPI. ***D***, Validation of TdTomato expression in the retinas of *Atoh1^FlpO/+^; Ai65F/+* mice. The retinas and cerebella were collected at P28 (*n* = 3). The nuclei were stained with DAPI. ***E***, ATOH1-GFP expression in the retinas of *Atoh1^GFP^*^/^*^GFP^* mice. The retinas were collected at P5, P7, P9, and P14 (*n* = 3 per timepoint). ***F***, The *t*-distributed stochastic neighbor embedding (tSNE) plot of the single-cell RNA sequencing data from P14 WT mouse retinas (Macosko et al., 2015). ***G***, Expression of *Atoh1*, *Pax6*, and *Vsx2* on the tSNE plot.

To eliminate the potential confounding effects of non-*Atoh1*-lineage neurons on phenotyping the *Atoh1* cKO mice, we used an intersectional approach to label the *Atoh1*-lineage neurons by crossing *mRx-Cre; Atoh1^FlpO^*^/+^ mice to *Atoh1^flox/+^; Ai65/Ai65* mice. The *FlpO* allele was knocked in to *the Atoh1* locus and replaced the entire coding region of *Atoh1*, resulting in an *Atoh1*-null allele (van der Heijden and Zoghbi, 2018). Using a Cre- and Flp-dependent TdTomato reporter *Ai65* allele (Madisen et al., 2015), we selectively labeled the intersectional domain of *Rax* and *Atoh1* in the retina (Fig. 4*A*). To our surprise, we did not observe any TdTomato^+^ cells in the retina using the intersectional approach (Fig. 4*B*). We confirmed individual recombinases were functional with the following experiments. First, *mRx-Cre; Ai14*/*+* mice demonstrated the efficient function of Cre recombinase shown by TdTomato labeling of all retinal cells (Fig. 4*C*). Second, we crossed the *Atoh1^FlpO^*^/+^ mice to *Ai65F* mice, which carry an Flp-dependent TdTomato allele. Although we observed TdTomato signal in the cerebellar granule neurons, a well established *Atoh1*-lineage neuronal population, no TdTomato^+^ cells were detected in the retina (Fig. 4*D*). These data were in stark contrast with the results from *Atoh1^Cre/+^; Ai14*/*+* mice (Fig. 1*B*). Given that Cre-dependent TdTomato reporter (*Ai14*) was in the *ROSA* locus with a strong CAG promoter, a trace amount of Cre recombinase is sufficient to drive TdTomato expression in the cells. Therefore, the presumed “*Atoh1*-lineage” neurons in the retinas of *Atoh1^Cre/+^; Ai14/+* mice may be artifacts caused by ectopic expression of Cre recombinase.

To further investigate whether *Atoh1* is expressed in the retinas, we performed immunofluorescence staining on the retina of a mouse model carrying *Atoh1-GFP* fusion allele (*Atoh1^GFP/GFP^*), which we have validated to mirror native *Atoh1* expression (Lumpkin et al., 2003). We collected the retinas from P5, P7, P9, and P14 mice and performed immunostaining using GFP antibody. At all timepoints, we did not detect ATOH1–GFP signal in the retina (Fig. 4*E*). In addition, we analyzed the publicly available single-cell RNA-seq data of mouse retinas at P14 (Macosko et al., 2015) and confirmed that *Atoh1* transcripts were not detected in all retinal cell types (Fig. 4*F*,*G*). Together, these data reveal an undetectable *Atoh1* expression in mouse retina, corroborating the finding that no TdTomato^+^ cells are observed in the retinas of *Atoh1^FlpO^*^/+^*; Ai65F* mice. Therefore, we conclude that there is an ectopic expression of Cre recombinase postnatally in the retinas in *Atoh1^Cre/+^* mice.

## Discussion

The Cre-loxP system has been widely used in genetic lineage tracing and cell type-specific genetic manipulation *in vivo*. The specificity and efficiency of targeting a particular cell type varies depending on the loci of the Cre, the loci of the targeted gene of interest, and the genetic background of the mice (Song and Palmiter, 2018). Indeed, several studies have revealed nonspecific Cre expression using transgenic or knock-in Cre lines (Hébert and McConnell, 2000; Spinelli et al., 2015; Wu et al., 2020; Ogujiofor et al., 2021). Therefore, careful characterization of the Cre knock-in mouse line is important for understanding the limitations and advantages of a mouse model.

*Atoh1^Cre/+^* mice have been widely used to target well established *Atoh1*-lineage cells including several neurons in the hindbrain and the spinal cord, hair cells in the inner ear, secretory cells in the intestine, Merkel cells in the epidermis, and tumor cells in the medulloblastoma. *Atoh1-Cre* expression in those lineages is validated given loss of the labeled cells on *Atoh1* knockout. In this study, we demonstrated the nonspecific labeling of the retinal cells in *Atoh1^Cre/+^; Ai14/+* mice by using *Atoh1^FlpO^*^/+^*; Ai65F/+* mice. The absence of the labeled retinal cells in *Atoh1^FlpO^*^/+^*; Ai65F/+* mice is not likely because of insufficient FlpO-mediated recombination in the retina given that *Atoh1-FlpO* allele successfully labeled all other *Atoh1*-lineage neurons shown in the present study (Fig. 4*D*) and a previous study (van der Heijden and Zoghbi, 2018). Moreover, other FlpO-mediated genetic tools have been used to study different cell types in the retina (Jo et al., 2018), disputing that FlpO recombinase may not function in the retina. Most importantly, we did not detect *Atoh1* expression in the mouse retina by immunostaining in this study (Fig. 4*E*) or in the published single-cell RNA-seq data (Macosko et al., 2015).

The mechanism underlying the ectopic labeling in the retinas of *Atoh1^Cre^*^/+^ mice remains unclear. However, the unexpected expression of Cre has been reported in the motor neurons of the intrinsic hand and foot using the same *Atoh1^Cre^*^/+^ mice with Ai14 reporter (Ogujiofor et al., 2021). Of note, Ogujiofor et al. (2021) and our study both demonstrated that the ectopic labeling happened postnatally and reliably in a particular group of cells across animals. These data suggest that the ectopic expression of Cre is not a stochastic event. Given the robust labeling of the glutamatergic amacrine cells, AII amacrine cells, and BC3b bipolar cells in *Atoh1^Cre^*^/+^*; Ai14/+* mice (Fig. 2), we propose to use the intersectional approach by combining *Atoh1^Cre/+^* mice and other FlpO mouse lines to achieve selective labeling of the retinal neurons. For example, *FlpO* driven by *Slc17a8* promoter in combination with *Atoh1^Cre/+^* can be used to target ∼89% of VGluT3^+^ amacrine cells in the retina, but not other *Atoh*1-lineage cells or other VGluT3-expressing cells in the brain. On the other hand, when targeting the retinal neurons is not desired, we suggest using *Atoh1^FlpO^*^/+^ mice rather than *Atoh1^Cre^*^/+^ mice since we did not observe the ectopic labeling in the retina in *Atoh1^FlpO^*^/+^ mice (Fig. 4*E*).

Although we did not detect *Atoh1* expression in the retinas or observe any histologic phenotype in the *Atoh1*-deficient retinas, a recent study demonstrated that overexpression of *Atoh1* and another bHLH transcription factor, *Ascl1*, in the retinal Müller glia stimulated neurogenesis in the adult mice (Todd et al., 2021). Importantly, Todd et al. (2021) also showed that endogenous *Atoh1* was not detected in adult retinas and that overexpressing *Atoh1* in the Müller glia alone is not sufficient to drive the glia-to-neuron reprogramming. Our data corroborate the previous study and demonstrate that *Atoh1* is dispensable for retinal development.

In sum, this study provides evidence that *Atoh1*, unlike its paralog *Atoh7*, is not expressed in the mouse retina. We also highlight the ectopic expression of Cre in the retinas of a commonly used mouse model, *Atoh1^Cre/+^*, which is important to note when interpreting data using this mouse line.

## Synthesis

Reviewing Editor: Eran Perlson, Tel Aviv University Decisions are customarily a result of the Reviewing Editor and the peer reviewers coming together and discussing their recommendations until a consensus is reached. When revisions are invited, a fact-based synthesis statement explaining their decision and outlining what is needed to prepare a revision will be listed below. The following reviewer(s) agreed to reveal their identity: Yanxin Pei, Jaleed Gilani.

This manuscript explores the expression and function of Atoh1 in mouse retinal development. First, Atoh1Cre knock-in mice were crossed with mice carrying a Cre-dependent TdTomato reporter, and Atoh1-FlpO knock-in mice were crossed with mice carrying a Flp-dependent TdTomato reporter. This enabled the tracking of Atoh1-lineage cells by identifying TdTomato+ cells within the retina. The researchers observed TdTomato-positive retinal neurons during the postnatal stage, including amacrine cells and bipolar cells in Atoh1-cre/TdTomato mice. However, TdTomato-positive retinal neurons were not observed in Atoh1-FlpO/TdTomato mice. Furthermore, they found no evidence of Atoh1 expression in the retina at various developmental stages, as demonstrated by immunohistochemical staining for GFP in Atoh1-GFP knock-in mice. In addition, the manuscript demonstrates that the knockout of Atoh1 did not impact mouse retinal development. As a result, the authors concluded that Atoh1 is not expressed in the mouse retina and is not essential for mouse retinal development. While it is a very well-written study, the authors need to provide evidence that FlpO recombinase function is not impaired within the retina of Atoh1-FlpO mice.

This study showed that Atoh1-lineage cells were present in Atoh1-cre/TdTomato mice but not in Atoh1-FlpO/TdTomato mice. The authors suggest that Atoh1-cre mice might exhibit ectopic Cre expression in the retina, leading to the presence of TdTomato+ retinal neurons in Atoh1-cre/TdTomato mice. However, an alternative explanation could be that FlpO recombinase function is impaired within the retina of Atoh1-FlpO mice. Therefore, an investigation into FlpO protein expression within the retina is warranted to shed light on this matter. Moreover, the distinct regulatory elements governing cre and FlpO expression in the retinas of the two knock-in mice might also contribute to the disparity in observing TdTomato+ cells in the retina.

Notably, a previous study demonstrated the presence of GFP expression in the retina of Atoh1-GFP transgenic mice (PMID: 12915300), warranting the discussion in the current study.

## Author Response

Reviewer #1:

While it is a very well-written study, the authors need to provide evidence that FlpO recombinase function is not impaired within the retina of Atoh1-FlpO mice.

Because FlpO allele was knocked-in to the Atoh1 locus, we do not expect any FlpO expression in the retina, if Atoh1 is not expressed there. FlpO is under Atoh1 promoter. This explains why we did not observe TdTomato-labeled cells in the retina of Atoh1FlpO/+; Ai65F/+ mice.

The absence of the labeled retinal cells in Atoh1FlpO/+; Ai65F/+ mice is not likely due to insufficient FlpO-mediated recombination in the retina given Atoh1-FlpO allele successfully labeled other Atoh1-lineage neurons shown in the present (Figure 4D) and previous study (PMID: 29972353). This should assure the referees that this knocked-in FlpO is intact and functional. Moreover, FlpO-mediated alleles have been used to study different cell types in the retina (PMID: 30186122), demonstrating FlpO is functional in the retina. Most importantly, we did not detect Atoh1 expression in the mouse retina by immunostaining in this study (Figure 4E) nor from the published single-cell RNA-seq data (PMID: 26000488).

We have added a paragraph in the discussion to address this concern.

Reviewer #2:

(a) However, an alternative explanation could be that FlpO recombinase function is impaired within the retina of Atoh1-FlpO mice. Therefore, an investigation into FlpO protein expression within the retina is warranted to shed light on this matter.

This is the same concern from the other reviewer. Please see our response above.

(b) Moreover, the distinct regulatory elements governing cre and FlpO expression in the retinas of the two knock-in mice might also contribute to the disparity in observing TdTomato+ cells in the retina.

Both Atoh1-Cre and Atoh1-FlpO knock-in alleles preserve the endogenous Atoh1 regulatory elements because only the open reading frame of Atoh1 (encoded by a single exon) is replaced with either Cre or FlpO recombinase. Both enzymes activate reporters in same cells. Therefore, the disparity in TdTomato-labeled cells in the retina is less likely due to the distinct regulatory elements in the two knock-in mouse models.

(c) Notably, a previous study demonstrated the presence of GFP expression in the retina of Atoh1-GFP transgenic mice (PMID: 12915300), warranting the discussion in the current study.

The GFP expression may not faithfully reflect the endogenous Atoh1 pattern in Atoh1-GFP transgenic mice (PMID: 12915300) for the following reasons. First, the transgenic mouse was generated by randomly integrating a fragment of DNA containing GFP reporter under a portion of the Atoh1 enhancer to the mouse genome. The expression of the transgene could be altered depending on the genomic locus it was inserted to. Second, the authors in that paper also acknowledged that GFP expression in the “non-Atoh1-related” domains (which include retina) is likely due to the absence of the inhibitory regulatory sequence from the transgene. Lastly, in addition to the retina, the authors also detected ectopic GFP expression in the cortex, dentate gyrus, and olfactory epithelium during embryonic stage. However, none of the regions were found to express Atoh1 in any other Atoh1 knock-in mouse models including Atoh1-Cre and Atoh1-FlpO mice used in our study, further highlighting the importance of using knock-in allele and the caveats of potential ectopic expression in the genetically modified mouse models.
