# Supplementary figures and images for: The Atoh1-Cre Knock-In Allele Ectopically Labels a Subpopulation of Amacrine Cells and Bipolar Cells in Mouse Retina
Source: eNeuro. 2023 Nov 2;10(11):ENEURO.0307-23.2023. doi: 10.1523/ENEURO.0307-23.2023 (PMC10626521; doi:10.1523/ENEURO.0307-23.2023)

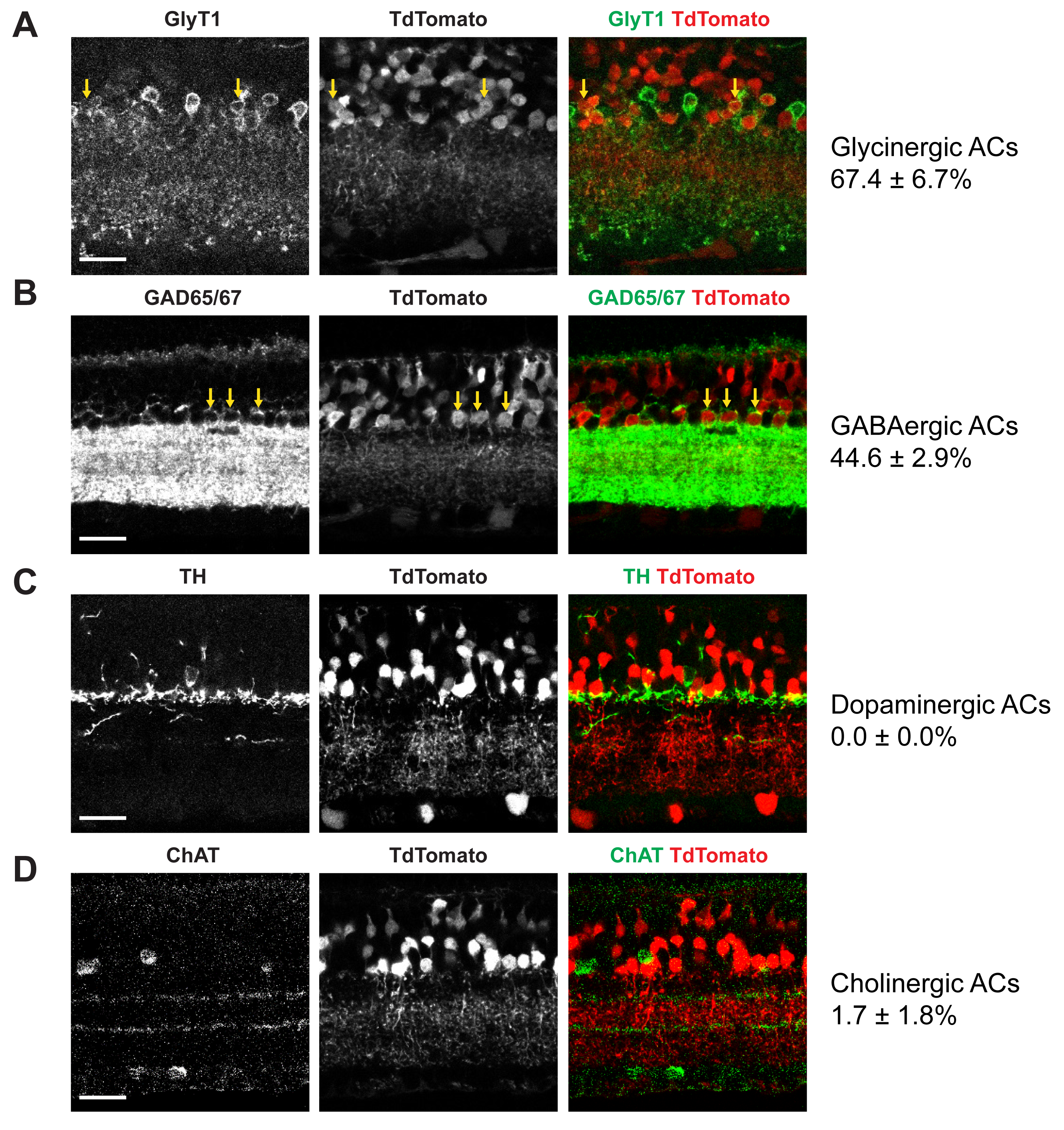

Supplement: Figure 2-1 — Characterization of TdTomato+ amacrine subtypes. A–D, Immunofluorescence staining of different markers for amacrine subtypes on adult mouse retinas. The retinas were collected from Atoh1Cre/+; Ai14/+ mice. The arrows show examples of the colocalization of the marker and TdTomato. The percentage of the marker+ cells overlapping with TdTomato is calculated and reported as the mean ± SD (n = 3 for each marker). Scale bar, 25 μm. ACs, Amacrine cells. Download Figure 2-1, TIF file. [file enu-eN-NRS-0307-23-s02.tif]

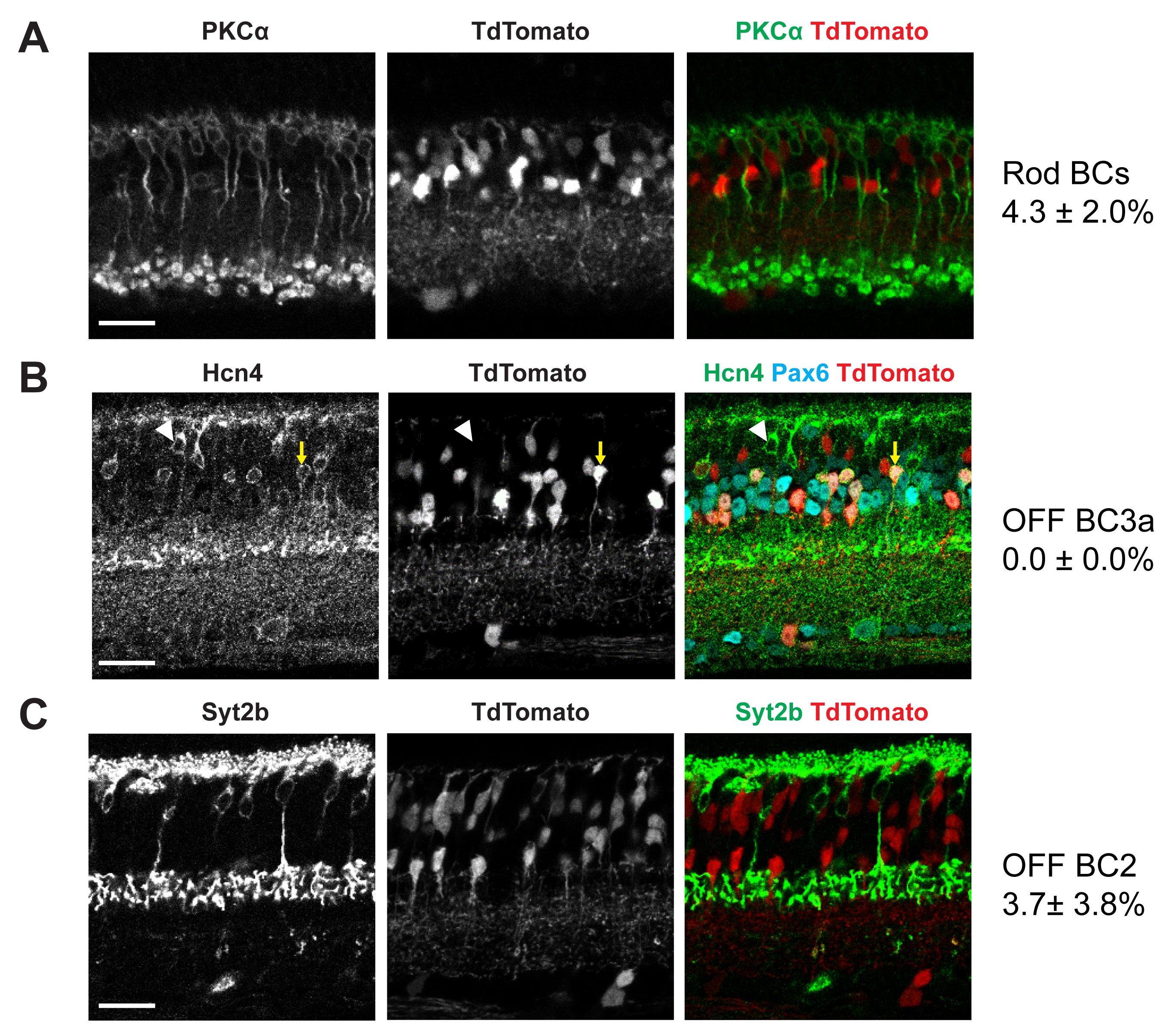

Supplement: Figure 2-2 — Characterization of TdTomato+ bipolar subtypes. A–C, Immunofluorescence staining of different markers for amacrine subtypes on adult mouse retina. The retinas were collected from Atoh1Cre/+; Ai14/+ mice. The arrows show examples of the colocalization of the marker and TdTomato. For BC3a subtype (B), Pax6 was used as an additional marker to exclude Hcn4+ amacrine cells, shown by the yellow arrow. In contrast, the white arrowhead denotes the BC3a neuron that was not labeled by TdTomato. The percentage of the marker+ cells overlapping with TdTomato is calculated and reported as the mean ± SD (n = 3 for each marker). Scale bar, 25 μm. BC, Bipolar cell. Download Figure 2-2, TIF file. [file enu-eN-NRS-0307-23-s04.tif]

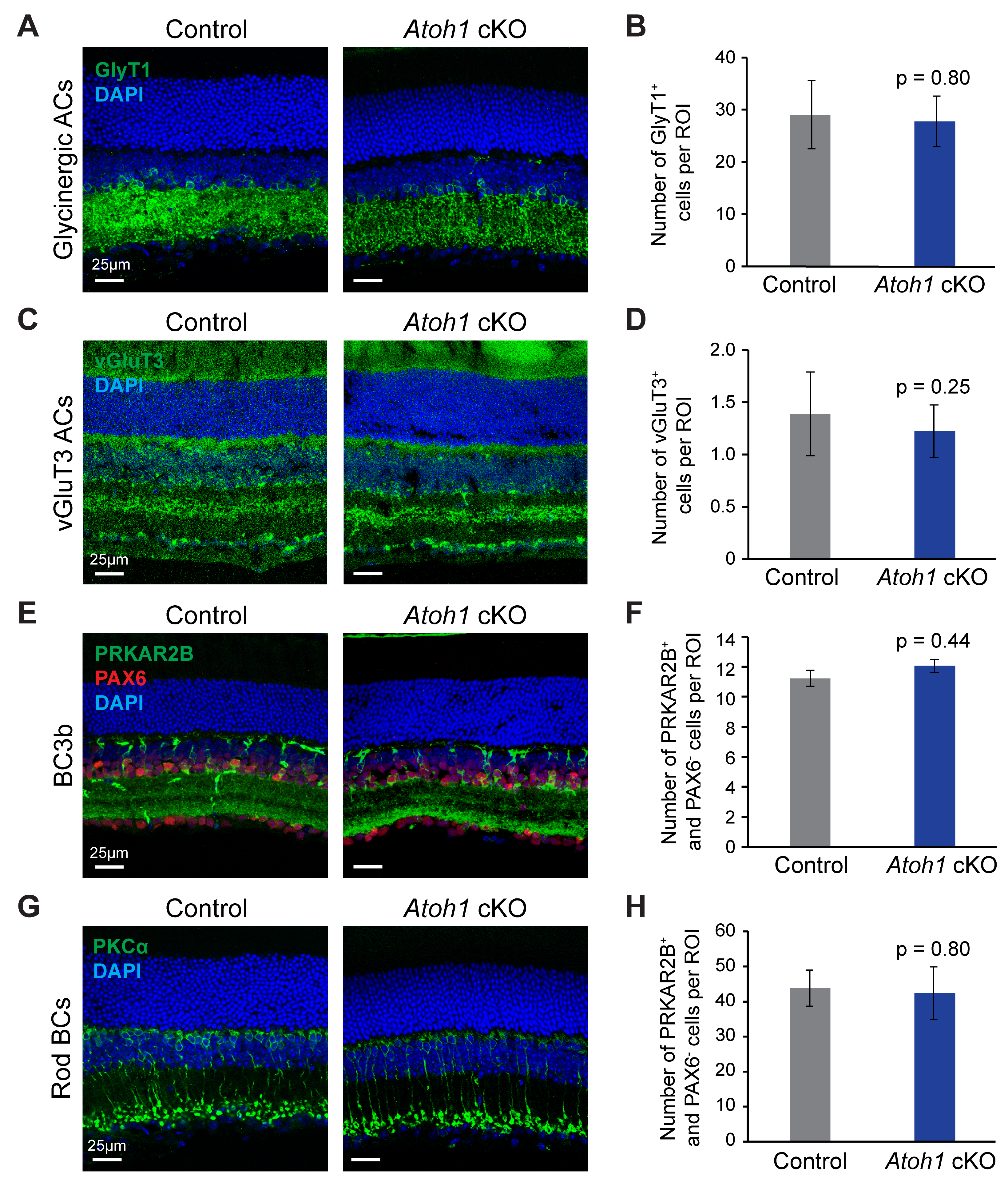

Supplement: Figure 3-1 — Atoh1 conditional knockout in the retina does not cause cellular phenotypes. A, C, E, G, Immunofluorescence staining of cell type-specific markers on the retinas from control and Atoh1 cKO mice (n = 3 per genotype). The nuclei were stained with DAPI. For BC3b subtype (E), Pax6 was used as an additional marker to exclude Prkar2b+ amacrine cells. B, D, F, H, Quantification of the retinal subtypes in control and Atoh1 cKO mice. Data are presented as the mean ± SD (n = 3 per genotype). The p values were determined by t test. Download Figure 3-1, TIF file. [file enu-eN-NRS-0307-23-s03.tif]
