# Supplementary material for: The Atoh1-Cre Knock-In Allele Ectopically Labels a Subpopulation of Amacrine Cells and Bipolar Cells in Mouse Retina
Source: eNeuro. 2023 Nov 2;10(11):ENEURO.0307-23.2023. doi: 10.1523/ENEURO.0307-23.2023 (PMC10626521; doi:10.1523/ENEURO.0307-23.2023)
Supplement: Extended Data Table 2-1 — Characterization the TdTomato+ amacrine cells in Atoh1Cre/+; Ai14/+ mice. Download Table 2-1, DOC file. [file enu-eN-NRS-0307-23-s05.doc]

|  |  | Glycinergic ACs | | | GABAergic ACs | | |
| --- | --- | --- | --- | --- | --- | --- | --- |
| Marker | % of TdTom^+^ | vGluT3 | AII | Others | Dopaminergic | Cholinergic | Others |
| Pax6 | 29.0 ± 3.1% | + | + | + | + | + | + |
| GlyT1 | 67.4 ± 6.7% | + | + | + |  |  |  |
| GAD65/67 | 44.6 ± 2.9% |  |  |  | + | + | + |
| TH | 0.0 ± 0.0% |  |  |  | + |  |  |
| ChAT | 1.7 ± 1.8% |  |  |  |  | + |  |
| vGluT3 | 88.8 ± 12.9% | + |  |  |  |  |  |
| Dab1 | 80.9 ± 7.1% |  | + |  |  |  |  |

**Table 2-1. Characterization the TdTomato^+^ amacrine cells in *Atoh1^Cre/+^; Ai14/+* mice.**

The first column lists the markers used to idetify the different populations of amacrine cells (ACs) on the right. The plus sign (+) denotes the subtype in which a marker is expressed. The second column lists the percentage of the marker^+^ cells that co-express TdTomato presented by mean ± standard deviation (n = 3 per marker). GlyT1, glycine transporter 1; GAD65/67, Glutamate decarboxylase 65/67; TH, tyrosine hydroxylase; ChAT, choline acetyltransferase; vGluT3, vesicular glutamatergic transporter 3.
