# Supplementary material for: The Atoh1-Cre Knock-In Allele Ectopically Labels a Subpopulation of Amacrine Cells and Bipolar Cells in Mouse Retina
Source: eNeuro. 2023 Nov 2;10(11):ENEURO.0307-23.2023. doi: 10.1523/ENEURO.0307-23.2023 (PMC10626521; doi:10.1523/ENEURO.0307-23.2023)
Supplement: Extended Data Table 2-2 — Characterization the TdTomato+ bipolar cells in Atoh1Cre/+; Ai14/+ mice. Download Table 2-2, DOC file. [file enu-eN-NRS-0307-23-s06.doc]

|  | | OFF BCs | | | | | ON BCs | | | | | |
| --- | --- | --- | --- | --- | --- | --- | --- | --- | --- | --- | --- | --- |
| Marker | % of TdTom^+^ | 1 | 2 | 3a | 3b | 4 | 5 | 6 | 7 | 8 | 9 | Rod |
| Vsx2 | 24.6 ± 4.3% | + | + | + | + | + | + | + | + | + | + | + |
| Islet1/2 | 8.7 ± 1.4% |  |  |  |  |  | + | + | + | + | + | + |
| PKCα | 4.3 ± 2.0% |  |  |  |  |  |  |  |  |  |  | + |
| Syt2b | 3.7 ± 3.8% |  | + |  |  |  |  |  |  |  |  |  |
| Hcn4 | 0.0 ± 0.0% |  |  | + |  |  |  |  |  |  |  |  |
| Prkar2b | 75.4 ± 5.0% |  |  |  | + |  |  |  |  |  |  |  |

**Table 2-2. Characterization the TdTomato^+^ bipolar cells in *Atoh1^Cre/+^; Ai14/+* mice.**

The first column lists the markers used to idetify the different populations of bipolar cells (BCs) on the right. The plus sign (+) denotes the subtype in which a marker is expressed. The second column lists the percentage of the marker^+^ cells that co-express TdTomato presented by mean ± standard deviation (n = 3 per marker). Vsx2, visual system homeobox 2; PKCα, protein kinase C-alpha; Syt2b, synaptotagmin 2b; Hcn4, Hyperpolarization Activated Cyclic Nucleotide Gated Potassium Channel 4; Prkar2b, Protein Kinase CAMP-Dependent Type II Regulatory Subunit Beta.
